# Navigating Discharge From Early Intervention in Psychosis Services: A Qualitative Exploration of the Experiences of Service Users and Carers

**DOI:** 10.1111/hex.70375

**Published:** 2025-08-10

**Authors:** Michelle Rickett, Tom Kingstone, Veenu Gupta, David Shiers, Paul French, Belinda Lennox, Ed Penington, Ryan Williams, Isobel Hoppe, Carolyn A. Chew‐Graham

**Affiliations:** ^1^ School of Medicine Keele University Keele UK; ^2^ Department of Psychology Durham University UK; ^3^ Department of Nursing and Public Health Manchester Metropolitan University UK; ^4^ Clinical Academic, Pennine Care NHS Foundation Trust, Department of Nursing and Public Health Manchester Metropolitan University UK; ^5^ Department of Psychiatry University of Oxford UK; ^6^ Division of Psychiatry Imperial College London UK

**Keywords:** discharge planning, early intervention, primary care, psychosis, qualitative methods, severe mental illness

## Abstract

**Introduction:**

Early Intervention in Psychosis (EIP) services in England offer up to 3 years' time‐limited support to people experiencing early psychosis. Service users (SUs) are discharged to primary care, a community mental health team (CMHT), or other specialist mental health service. The aim of this study is to explore the SU and carer journey through discharge from EIP and into the early post‐discharge period.

**Methods:**

Qualitative longitudinal study comprising semi‐structured interviews with SUs and carers at, or shortly after, discharge from EIP, and follow‐up interviews with SUs 6‐11 months later. Data collection conducted between January 2023–September 2024 and informed by information power. Data were thematically analysed by a multidisciplinary team.

**Results:**

SUs and carers expressed their desire to be actively involved in EIP discharge planning and decision‐making. They contrasted close relationships with EIP practitioners with inaccessibility of care and difficulties navigating healthcare systems after discharge. Some SUs described feelings of abandonment and expressed a wish for transitional support, and proactive, relationship‐based care post‐discharge. Carers played an important role as patient advocates but were rarely offered support themselves.

**Conclusion:**

Improved collaboration is needed between SUs, carers and primary care/CMHT practitioners in the build‐up to EIP discharge. There should be proactive contact from primary care at the point of discharge and in the early post‐discharge period. Carer needs are often overlooked; primary care could utilise the ‘carers register’ and proactively offer support.

**Patient or Public Contribution:**

Patient and carer involvement and engagement was key to all stages of this study. The research team met regularly with our two co‐investigators with lived experience (as a service user and a carer), who contributed to data analysis and writing this paper. We worked closely with our patient and carer advisory group, EXTEND‐ing, throughout the research process. They helped formulate research questions, co‐designed topic guides and participant information sheets, and contributed to data analysis and interpretation.

## 1 Introduction

Early Intervention in Psychosis (EIP) services offer multidisciplinary treatment in the community to people experiencing a first episode of psychosis (a severe mental illness characterised by hallucinations and delusions). In the United Kingdom, EIP is a time‐limited service of up to 3 years' duration. Service Users (SUs) and family members are offered a treatment package including case management with a specified ‘care coordinator’, pharmacotherapy, cognitive behavioural therapy for psychosis (CBTp), family intervention, physical health assessments and interventions, employment and education support, and carer support. EIP services are designed to reduce the duration of untreated psychosis and have been shown to improve outcomes such as symptom severity and general functioning engagement with mental health services, hospitalisation rates [1, 2, 3].

EIP services are reported to be highly valued by SUs both for the quality of therapeutic relationships established, particularly with their care‐coordinators, and for the range of treatments offered [4, 5]. Good communication and partnerships between healthcare practitioners, SUs and carers are reported as essential features underlying the successful delivery of EIP care [6]. However, there is little guidance around planning and implementation of discharge from EIP to other services, particularly to primary care, where the majority of people will be discharged without any ongoing specialist mental health input [7, 8]. There is reported to be a lack of collaboration between EIP and other healthcare services, particularly primary care, in the lead up to discharge [9, 10, 11].

Family members play a key role in supporting people with severe mental illness, both in terms of ‘looking after’ their practical needs and ‘caring for’ their emotional needs [12]. Strong relationships and empathetic engagement between healthcare professionals and carers are considered essential to effective care partnerships [13]. However, carers of people with early psychosis can experience substantial distress, impacting on their own health and wellbeing [14, 15]. Previous research suggests that the needs of carers can be overlooked by health services, which is compounded by a tendency for carers not to seek help for themselves [16].

The aim of this qualitative study was to explore the SU and carer journey through EIP discharge and post‐discharge care, focusing on their engagement with healthcare practitioners, involvement in decision‐making, and perceived gaps in support. The data included in this paper is part of a larger qualitative data set, which also included interviews with EIP healthcare practitioners, GPs and commissioners [17].

## 2 Methods

This qualitative longitudinal study comprised in‐depth semi‐structured interviews, exploring the views and experiences of EIP SUs and carers. Our methods were underpinned by interpretivism [18] to support exploration of contexts, meanings and interactions in relation to EIP.

### 2.1 Patient and Public Involvement (PPI)

Patient and public involvement (PPI) was integral to all parts of the study. We held regular meetings with our two PPI co‐investigators (VG, DS) and our patient and carer advisory group (EXTEND‐ing), who co‐designed topic guides and public‐facing documents, advised on recruitment strategies and contributed to data analysis and writing.

### 2.2 Recruitment of SUs and Carers

We recruited EIP SUs (18 years and above) at the time of, or shortly after, discharge from an EIP service. SUs that lacked capacity to consent or expressed suicidal ideation were excluded. We recruited carers of SUs at the time of, or shortly after EIP discharge, with no exclusions. Participants were recruited using both purposive and convenience sampling. Clinicians at EIP teams in six Mental Health Trusts (MHTs) around England were approached and asked to identify suitable participants. Additional participants self‐identified by responding to a study flyer shared via social media (X), mental health networks, support groups and charities. The flyer included a QR code linking to the study website.

After reviewing an information sheet, participants recruited via MHTs returned a ‘consent to contact’ form by post or email, after which a study researcher contacted them to confirm eligibility and arrange their preferred mode of interview (online via Microsoft Teams or by telephone). Participants responding to the social media flyer followed a similar process, beginning with an email exchange, then a screening call, to confirm capacity and inclusion criteria, and help exclude ‘imposter participants.’ [19] SUs could choose to be interviewed with their carer or not, and vice versa.

Consent forms were completed electronically before or at the start of the interview. At the end of each interview, participants were offered a shopping voucher as reimbursement and could opt to receive a summary of findings and related publications.

SUs were asked for permission to be contacted 6 months later for a follow‐up interview. Those who agreed were contacted, received an updated information sheet, were telephoned to confirm capacity, re‐consented, and participated in follow‐up interviews conducted under the same conditions as the initial interviews.

### 2.3 Data Generation

In‐depth semi‐structured interviews were conducted by the first author (MR), a female post‐doctoral researcher and social anthropologist with qualitative research expertise. Interview participants were not given any information about the researcher other than the university she was employed by.

Topic guides for both SUs and carers explored experiences and views of EIP services; duration of care; discharge planning and decision‐making; and plans for further care. A topic guide for SU follow‐up interviews focused on SU experiences after discharge; support accessed; gaps in support; and additional reflections on care received under EIP. (Please refer to ‘Supplementary File: Topic Guides’ for further information.) All topic guides were produced by the research team in collaboration with people with lived experience as EIP service users and carers, and were modified iteratively alongside data generation and analysis [20, 21]. Data collection was informed by information power; this is an alternative concept to saturation in qualitative research and involves pragmatic judgements based on aims, specificity, theory, dialogue and analysis [22]. Interviews were conducted between January 2023 and September 2024. The researcher (MR) also compiled fieldnotes after each interview to aid analysis.

The interviews were audio‐recorded, transcribed by a professional transcribing company, then checked and anonymised by the first author (MR). Analysis was performed using qualitative research software (NVivo 11). Transcripts were not returned to participants for comment and participants were not asked to give feedback on the findings. A thematic analysis approach was applied, led by the first author; this involved the researcher reading and rereading transcripts, identifying and organising codes, and constructing themes [23]. Data from individual participants were constantly compared across the data set to identify shared experiences, points of difference and to further refine codes and themes. MR coded the data, led analysis and met regularly with research team members (CCG, TK) and authors (VG, DS), with a range of backgrounds and disciplines, to check data interpretation and agree on themes.

The EXTEND‐ing patient and carer advisory group also helped to identify, clarify and deepen the research themes during analysis focused meetings with the research team and via email feedback. Several EXTEND‐ing members wrote reflective pieces about their own journeys through EIP after a meeting held to discuss SU and carer transcripts, which resonated with our analysis but were not used as research data.

## 3 Results

Twenty‐eight first interviews were conducted with 16 EIP SUs and 14 carers lasting 25–50 min. Two interviews were dyadic (joint SU/carer). Fourteen SUs were recruited through MHTs and two through SU groups. Three carers were recruited through MHTs, five through carer groups and six through social media. Twenty‐six people replied to our social media SU and carer recruitment flyer but either did not reply to follow up contacts, or, following a screening call (conducted by MR), were found to be ineligible as they had not been under EIP care, or were excluded as they were unable to answer screening questions about EIP care (likely ‘imposter participants’) [22].

All SUs who had consented to be contacted for follow‐up interviews agreed to participate. Twelve follow‐up interviews were conducted, lasting 20–40 min: eleven of these were conducted 6‐8 months after first interview and one SU was re‐interviewed for a second time at 11 months post‐discharge, as she had been waiting to be discharged from EIP to a CMHT at first follow up. Two of the follow‐up interviews were dyadic (joint SU/carer).

Participant characteristics are reported in Tables 1 and 2. Nine SUs were unemployed and thirteen were discharged to primary care.

**Table 1:** SU demographics.

Participant ID	Gender	Age	Ethnic background (self‐described)	Living circumstance	Second interview
SU1	Female	32	White British	Lives with partner and children	✓ (Reinterviewed twice as SU was still waiting to be discharged at first follow up)
SU2	Female	28	White British	Lives alone	✓
SU3	Female	28	White and Black Caribbean	Lives with partner and child	✓
SU4	Female	26	Mixed/multiple ethnicity	Lives with father	✓
SU5	Female	43	White Polish	Lives with partner and child	✓
SU6	Female	28	White British	Lives with father	✓
SU7	Female	61	White British	Lives with partner	✓
SU8	Female	57	Black Caribbean	Lives alone	✓
SU9	Male	43	Black British	Lives with partner	⌧
SU10	Female	64	White British	Lives with partner	⌧
SU11	Male	25	White British	Lives alone	✓
SU12	Male	43	White British	Lives with partner and children	⌧
SU13	Male	45	White British	Lives alone	✓
SU14	Male	32	White British	Lives with parents	✓
SU15	Male	31	White British	Lives alone	⌧
SU16	Male	50	White Australian	lives with partner	⌧

**Table 2:** Carer demographics.

Participant ID	Gender	Relationship to person with Psychosis	Age	Ethnic background (self‐described)
CAR1	Male	Brother	25	Asian
CAR2	Male	Friend	26	Black British
CAR3	Female	Niece	25	Indian British
CAR4	Female	Mother	65	White British
aCAR5	Male	Husband	59	White British
CAR6	Male	Grandchild	23	Black British
CAR7	Female	Mother	59	White British
CAR8	Male	Partner	65	White British
CAR9	Female	Former partner	45	Bangladeshi
CAR10	Female	Mother	45	White British
CAR11	Female	Mother	41	White British
CAR12	Female	Mother	58	White British
aCAR13	Female	Mother	69	White British
CAR14	Female	Daughter‐in‐law	39	White Other (Italian)

^a^ Dyadic interviews with SU.

Our findings are reported under two key themes: approaching discharge; post‐discharge needs and challenges. Illustrative data are presented to support the analysis along with identifiers (e.g., SU3).

## 4 Approaching Discharge

The theme ‘approaching discharge’ relates to the SU and carer journey as they come towards discharge from EIP. Sub‐themes explore SU and carer views on and experiences of *involvement in planning and decision making; anticipating abandonment; ongoing care—planning and transition; opportunities to build connections; carer role and expertise in relation to discharge planning*.

### 4.1 Involvement in Planning and Decision‐Making

All SUs expressed a desire to take part in decision‐making and planning around their discharge from EIP services:

That transition should be definitely in there where kind of everybody works together, everybody is equal, and then I feel like as a patient you should kind of be like, ‘right, I am ready now’ as opposed to, ‘right, there you go, see you later, bye’, you know, like a bit more patient involvement as opposed to, ‘you only get 3 years and then you're going to go to something completely different’.

(SU3)

There were two contrasting narratives about how this had worked in practice, with some SUs and carers feeling actively involved in discharge planning and decision making and others reporting lack of collaboration and feelings of disempowerment.

In cases where SUs felt involved in decision making about their discharge, they reported positive experiences of the transition from EIP:

I think having a discussion with my care coordinator, and a chat yesterday when I had my consultant appointment, it was just a joint view, really. It's all been coordinated and I've had my decision in that.

(SU1)

In one case, the duration of EIP care was extended beyond 3 years as a result of collaborative decision‐making between the SU and EIP practitioners. The SU valued their active involvement in this process:

I felt that they did listen to me and responded very appropriately and very positively.

(SU16)

Even when the decision about the timing of discharge was EIP practitioner‐led, good communication and tailored planning could help SUs feel supported and more positive about the future:

I've been in contact with my CPN and everything and they've been making sure that I'm not getting too overwhelmed about it. I have been able to ask questions. I'm not really good with change, so they've been taking it slow with us, which I'm really grateful for… I didn't feel like pushed off a cliff or anything…. It felt more like, ‘Here's the target and we'll help you get there’.

(SU6)

A minority of carers reported that they had been well informed throughout the discharge process:

It was discussed wasn't it with the consultant and we were always in the loop with everything that was said and done.

(CAR13)

In contrast, some SUs and carers reported that they had not been involved in discharge decisions or planning. SUs reported feeling disempowered when discharged before they felt ready, particularly to primary care rather than CMHTs. The decision to discharge seemed to be driven by the 3‐year time limit, which forestalled any negotiation, and led to SUs experiencing any discussion as tokenistic and inauthentic:

And, given the choice, I would still have been with them if I could've been. I found them very, very helpful. But it was the end of the 3 years.

(SU7)

They just, you know, they couldn't keep me longer so, that was reason, yeah. So, they asked me if I agree with that, I said, ‘yes,’ I didn't have a choice I think, you know.

(SU5)

Some carers reported that they and their loved ones did not play an active role in decision‐making and felt pressured into discharge, leading to negative experiences of transition.

She's just a bit of flotsam in the system as far she's concerned, I think. I mean they did try and involve her in all decision‐making but when the decision is—‘You're leaving us and you're going to the CMHT,’ and there's no decision to be made and there's no choice—you can flannel and you can dress it up, but it is what it is. So, in a way, it was not particularly easy at all and, in fact, it was a very bad transition in my opinion.

(CAR4)

### 4.2 Anticipating Abandonment

While under EIP care, SUs had developed strong and trusting relationships with EIP practitioners (particularly their care coordinator) but reported little to no contact with primary care for either mental or physical health.

The GP practice don't know an awful lot really, I think it's good that the hospital (EIP service) keeps you there quite a few years, I think it's nice really, you don't have to deal with your normal GP. Because like I said I don't actually know who my GP is…

(SU2)

I do know a copy (of the discharge plan) goes to your GP, but I have not had any direct contact with my GP regarding my mental health.

(SU3)

SUs and carers expressed a sense of abandonment about the impending loss of EIP relationships. This was experienced both by those being discharged to primary care and those being transferred to CMHTs.

Personally, I'm terrified (of discharge) because all my help I know where to go, I know where to get the help immediately if I need it. Any problems I know what to do and I know his care coordinator so well that I feel comfortable.

(CAR7)

(There's) this sense of being bereaved and a sense of loss. And that's not just from a support system that understands you and you don't have to explain everything to, but obviously an individual aspect of that which is the relationship and rapport we've built with the person that has worked alongside, well effectively like coproduction isn't it for that many years.

(SU10)

### 4.3 Ongoing Care: Planning and Transition

Three of the 16 SUs were discharged to CMHTs. Joint handover meetings with CMHTs before discharge were planned but didn't always happen in practice:

(CMHT care coordinator) was going to come to my hospital appointment but she couldn't make it, so I think she had something else on, so they did try to meet together, but it just didn't work out.

(SU2)

Before their discharge to a CMHT, these SUs expressed apprehension and lack of clarity about their ongoing care:

I am very, very anxious regarding the level of availability, the treatment, the familiarity, the resources, the help that I am going to receive from the CMH team so, again, like is it a trigger? Is it not going to be enough? Is it going to be the right course of treatment even though they are there?

(SU3)

The majority of the SUs were discharged to primary care. Most of these SUs reported that they had crisis plans and contact details for ongoing support:

I got given a plan, a care plan. So it has all the stuff on there. You know, like crisis team stuff and what to do when I'm having a meltdown type thing.

(SU6)

However, they also expressed concern about having to re‐tell their story following discharge to GPs that they hadn't developed or maintained a relationship with:

What I have asked is although I've got a named GP at my doctors, it has changed where it can be multiple people that you've not really seen before. So, I did ask my consultant yesterday if I could… I think for me, it's about someone who just has a little bit of an idea about what's gone on previously…. but it's a bit annoying, I think, especially when you are not feeling good mentally, when you're having to repeat yourself and tell your story time and time again.

(SU1)

For some, discharge to primary care felt like moving on without support:

…now I feel a bit lonely you know, you just have to care about yourself on your own, that's it, yeah.

(SU6)

One carer felt that a handover meeting between EIP and primary care professionals at point of discharge could help the transition and encourage a more personalised approach:

I don't know if it's possible for anything like that, but I think, could that psychiatrist have a meeting with his new doctor, (*GP*) and share what they've learnt about (service user) and how to best care for him.

(CAR10)

Another carer suggested that SUs should be seen by a GP soon after discharge as a matter of course:

When patients are discharged under the mental health team they have to be reviewed within 72 h by someone somehow and that's mandatory. So I think there should be something like that, like an algorithm type thing that the GP also must see them within a week or two and then at least they'd see them after discharge.

(CAR13, follow up)

In some cases, SUs balanced their sense of loss of EIP services against awareness of the progress they had made:

I guess I am a bit afraid about what comes next because it does kind of feel like somebody has took the lead off, kind of thing. You won't be able to talk to the people like you've formed a connection with for 3 years and that's kind of scary. But you know, I feel like I have the tools to deal with it than I did 3 years ago.

(SU6)

I was slightly sad though because I'm really close to my nurse so it was going to be a bit strange. But I'm actually really proud of how far I've come and proud of the progress that I've made and I'm just heading into the future.

(SU4)

### 4.4 Opportunities to Build Connections

Some SUs had participated in EIP‐led support groups and valued these more informal engagements with other SUs and practitioners:

Something to do, didn't have to pay for it, friends there with similar problems.

…And there were medical professionals there as well, so it's a good informal environment to chat to them. Sometimes a nurse would go, sometimes a psychologist would go, but someone would go each time as well as the physical health worker.

(SU11)

They expressed a desire to continue this kind of engagement after discharge through being sign‐posted to third sector organisations:

It would've been nice to be offered like connections to like support groups, to like social prescribing type things. I think that'd be really beneficial.

(SU3 follow up)

Some SUs talked more positively about discharge if they had been informed about groups that they could join:

I'm not on my own…. And there's a charity that I can get involved in, (local mental health charity), which help people who have had mental health issues, that you can get involved in all sorts of groups, gardening groups, yoga, meditation. So, you know, there is support out there.

(SU8)

### 4.5 Carer Role and Expertise in Relation to Discharge Planning

Carers often described taking on a ‘case manager’ role during and after discharge, due to their knowledge of their loved ones’ particular needs and perceived gaps in their care.

I will get in touch with his GP about it as well…see if the GP is able to refer him to anyone about his addiction and alcohol abuse, someone outside of the EIP team.

(CAR3)

Some carers described feeling disempowered if their concerns were not listened to by healthcare professionals:

Her weight is a massive issue and chaos of eating. Who knows what her heart rate is? She needs beta‐blockers now but nobody is monitoring that and they're just re‐prescribing. Does she need them? Does she continue to need them? I don't know

(CAR4)

When I last met with Dr (psychiatrist) and (care coordinator) and (nurse) I asked that question again, ‘Will he go to the GP, or …?’ because I know that was always the plan, but (SU) has been slightly unstable recently, I didn't know if it would be worth him going to the adult mental health team, but they said, ‘Oh no, he's quite stable, so he'll go to the GP.’

(CAR10)

Carers of SUs with continued symptoms and high level of need expressed concern about the transition from intensive support under EIP to lower‐level support:

If somebody's got a diagnosis of schizophrenia, which is lifelong, that's very different to somebody that's experiencing a one‐off psychosis. She needs long‐term support. Which I'm obviously very worried about is going to be even worse when she's not under EIP and part of a community team.

(CAR11)

Some carers felt that their loved ones were reluctant to express their feelings about discharge to healthcare professionals. In such instances, carers could have an important role in communicating SU need:

‐ So, in the meeting that you had, did she say that she was concerned, worried?

(INT)

‐A little bit. It was me rather than (SU), because (SU) also feels she doesn't want to be an encumbrance. She doesn't want to do that, so she's more likely to say, ‘Oh I understand, thank you very much’ to be seen as polite and formal. But inside, in turmoil.

(CAR8)

Carers also said that there could be a disconnect between what they and the SU would communicate when the SU was ill, and were concerned that their views may not be listened to by healthcare professionals following discharge:

I should say to (care coordinator), actually, and the psychiatrist, can they put in a letter to the doctor's…that (SU) will say that he's fine, and we'll be saying something else. Because (SU) has said that to them before, as well…For whatever reason, he doesn't really know why, he doesn't present as that unwell, and he doesn't really seem to have the insight of actually what he is doing, his reality is a bit distorted to actually what is going on. So, for a new doctor, I just feel like we might be in the same position of, ‘Oh, (SU) is really not well,’ ‘He's not sleeping, he's doing …’ you know, X, Y, Z, and (SU) will just be saying, ‘No,’ ‘I'm absolutely fine.’ ‘They don't know anything.’…So I don't feel very confident, in terms of what it's going to be like.

(CAR10)

‘Approaching discharge’ highlights varying SU and carer experiences of shared decision decision making and planning around EIP discharge. It illustrates the importance of the relationships developed between SUs/carers and EIP healthcare practitioners, as well as concerns about the ending of these relationships and ongoing care needs. This theme also highlights the vital but under‐utilised role of carers in the discharge process.

## 5 Post‐Discharge Needs and Challenges

The theme ‘post discharge needs and challenges’ explores SU and carer experiences of healthcare in the months after discharge from EIP. Sub‐themes are *forming new relationships with healthcare practitioners; recognising problems and re‐accessing specialist support; support for carers*.

### 5.1 Forming New Relationships With Healthcare Practitioners

SUs and carers expressed a desire for regular care and/or transitional care after discharge and frequently described feeling alone and unsupported:

I think if I'd seen a healthcare person (after discharge) maybe just once a month just to clear up some questions that we had when we didn't have the hands‐on help, if you know what I mean. We were left second‐guessing, weren't we, really?

(SU7 follow up)

We didn't have any follow‐ups particularly from the GP after he was discharged from the EIP. And of course now we're back into the system for now anyway (following relapse)

(CAR13 follow up)

The experiences and outcomes of SUs discharged to CMHTs were dependent on the quality of relationships developed with CMHT care coordinators. This dictated whether the feelings of abandonment expressed before discharge continued to be felt in the post‐discharge period. Two SUs developed good relationships with new care coordinators and reported that they still received regular, personalised support:

So, I've got someone called (name—CMHT care coordinator) and she comes round every 2 weeks to my house to see if I'm okay. She helps me out with anything that I need, like any forms or anything like that.

(SU2 follow up interview)

In my experience it's basically been the same sort of care, I've received the same sort of care from both teams. This team has been a little bit less hands‐on….I've been a bit more independent under it, but yeah, it's basically the same.

(SU15)

However, another SU (who had expressed concerns and feelings of abandonment at point of discharge) was unhappy with the support she had received from her CMHT, was then discharged from CMHT to primary care, and felt she hadn't been able to build any trusting relationships with any healthcare professionals after discharge:

I just feel like I'm being passed from post to post now and different people all the time. There's no consistency and it's gaining, building that trust.

(SU3 follow up)

SUs discharged to primary care compared the easy access to care they had experienced under EIP with lack of contact with, and inaccessibility of, general practice:

I would like to have a chat every 6 months as before with the psychiatrist and it was only 20 or 15 min, but you know, he always said something that we were going to do if the voices won't stop, he would put me on another medication or I don't know, but now, it's nothing, completely nothing, yeah.

(SU5 follow up)

It's hard to get an appointment. It's hard to speak to somebody; whereas, before, I could just phone the team and there would be somebody to speak to.

(SU7 follow up)

The contact that SUs did have with primary care was described as limited:

All I've seen is a nurse twice since I've been with that surgery and that was for my blood test. So they have to check me once a year for the medication. She's the only person I see. I've never seen any of the doctors. I don't know who they are or what they look like.

(SU13)

In one case, an SU who had expressed feelings disempowerment and abandonment at point of discharge, reported a limited and depersonalised response from primary care when she began to experience symptoms again after discharge:

I called my GP once when the voices started being very rude to me and he increased my dose so that was one time….I just did it over the phone, that's it.

(SU5)

In some cases, negative experiences could lead to SUs ‘giving up’ on trying make contact with their general practice:

I've sort of given up with (making GP appointment)…. I never see them face to face; I may get a phone call, at best. And even something as simple as getting my blood tests results back took probably more like two, two and a half weeks, and they kept telling me, ‘If there was a problem, we would've contacted you,’ but every time I rang them up for my results, they just kept saying that the doctor hasn't checked them yet…and then, all of a sudden, I get messages saying, ‘You've got high potassium, you've got high cholesterol,’ so no one had looked at them.

(SU13 follow up)

Carers confirmed that SUs could decline to access primary care following discharge about physical health problems due to a perceived lack of established relationship and trust:

She had a swollen cheek 3 weeks ago and I said, ‘You can't leave that.’ She was saying, ‘My teeth hurt,’ but then she said, ‘Oh, it will be better.’ So she hasn't been and I said, ‘Shall we get the GP?’ ‘No, no, I don't want to see a GP.’ No, there is almost no relationship with them.

(CAR4)

In one case, a SU described being contacted by her GP at point of discharge and put in contact with a mental health practitioner within the practice which helped the SU feel cared for:

The GP got in contact with me to make sure that I go to this appointment with, I think she's a mental health nurse or something…and she just went through everything with me, and she made me feel like everything was going to be okay.

(SU6 follow up)

### 5.2 Recognising Problems and Re‐Accessing Specialist Support

SUs and carers anticipated difficulties re‐accessing specialist services post‐discharge. The lack of relationship with primary care meant that they felt they would have to navigate services without support.

I think that's my only concern is it's easy to be fine with it when you're feeling okay, but because of how seriously unwell I was, there is that concern of like, if I did have a relapse how easy would it be to, would the GP know? Do you know what I mean?

(SU1 follow up)

My first port of call would be the GP. I wouldn't know who I would be speaking to there…And I would feel like we have to go back to square one again and start again. And so I would be doing that out of desperation with no confidence that there would be an appropriate reaction.

(CAR8)

SUs who needed to reaccess specialist support after EIP discharge found this a challenging and stressful experience:

The early intervention service told me I could refer myself [back to specialist care] and you can't, it turns out,,, I kept ringing up mental health teams and they kept telling me I had got to call another team because of where I live…I called them up for like 2 hours and I got really annoyed and then, finally, I got through to the right (team) then and “you need to get your GP to refer you, you can't do this”, and I was like, ‘that's great’. My mum did it for me in the end because I got too annoyed, and I wasn't feeling well at the time.

(SU11 follow up)

A minority of SUs were proactive about seeking support from specialist NHS or third sector services:

I'm engaging with Healthy Minds because I know how quickly, even when you feel okay then it could, you know, it can quickly change. I'm just very conscious that I'll always have mental, you know, be fragile to mental health, shall we say. But yeah, largely recovered.

(SU1 follow up)

So, in November I contacted the GP to say that I had actually self‐referred to talking therapies.

(SU10)

In some cases, SUs felt that they had little choice but to pay for private support following discharge.

I am now paying for a psychologist…I wasn't part of the mental health team at the time…and I didn't see any way of speaking to one really.

(SU11)

### 5.3 Support for Carers

None of the carers interviewed were offered proactive support by healthcare professionals during or after their loved ones’ discharge from EIP. Carers seemed to underplay their own needs and not access support for themselves:

At the time if it had been offered, I would've probably replied, ‘I'm okay, I don't need it.’ Now, looking back, I knew I did….I'm aware that the way I supported (SU) was by parking myself completely. And side‐lining any feelings I had, and I don't think that has been beneficial.

(CAR8)

One carer had proactively accessed private counselling support in the past and expressed willingness to do this again:

In the future, maybe I might maybe reach out for some counselling or something, which I did do when (SU) was first ‐ the first year I paid for private counselling, just to… I just needed someone to talk to and really to listen to what was going on and they weren't… Yeah, it was just helpful to have somebody that understood those sorts of things.

(CAR10)

Another carer had set up her own carer support group in response to lack of statutory provision:

I've got in contact with (mental health organisation), and (met) 3 women … and we've set up a Carers’ Support Group local to us here now. So, we've kind of created our own support.

(CAR12)

‘Post discharge needs and challenges’ illustrates a lack of relationship‐based care for most SUs and carers after discharge from EIP, which intensifies the sense of abandonment that they felt earlier at the prospect of losing relationships with EIP practitioners. It highlights difficulties in re‐accessing specialist support when SUs need it and a lack of post‐discharge support for carers.

## 6 Discussion

### 6.1 Summary

This longitudinal study illustrates how discharge from EIP services and the early post‐discharge period can be particularly challenging for SUs and carers. Our findings highlight the importance of the close relationships SUs and carers build with EIP practitioners, and the consequent feelings of abandonment they can feel in relation to discharge, particularly if they feel excluded from decision‐making or unprepared for the transition. Discharge experiences were more positive when SUs and carers perceived involvement in planning as genuine and where they were signposted to post‐discharge support networks and organisations.

Our findings highlight SU and carers’ desire for a better managed transition of care post‐EIP. Some SUs expressed frustration about challenges in accessing primary care following discharge and some participants reported using community and voluntary organisations and private providers for additional support. Both SUs and carers expressed concerns about their ability to re‐access specialist support if needed.

Carers play a key role as advocates during the discharge period, informed by their understanding of their loves ones’ needs, symptoms, triggers, and treatment histories. Carers can ‘fill the gap’ in navigating healthcare systems as SUs transition to onward care. However, our findings suggests that carer expertise is not perceived to be valued by healthcare practitioners, and carers are not offered support by onward healthcare practitioners after EIP discharge.

### 6.2 Comparison With Existing Literature

Our paper adds to previous limited research into SU experiences of discharge from EIP. Two previous qualitative studies highlight the sense of loss that SUs can feel when moving on, particularly from EIP therapeutic relationships; the importance of SU perceived ‘readiness’ for discharge and advance planning; and gaps in post‐discharge care [24, 25]. Our research is consistent with these findings and also highlights the value SUs attach to being actively involved in discharge decision‐making and how this impacts on their experiences. We also include the views and experiences of carers, who play an important and challenging role in supporting the needs of people suffering from psychosis [26, 27] This is a perspective missing in previous research on experiences of EIP discharge. Our research shows the key role that carers play in the discharge process, as advocates for SUs and by taking on the role of a care coordinator.

Research has shown that the continuity of care provided within EIP services—particularly the relationship with care coordinators—is highly valued and leads to improved outcomes [28, 29]. In general, continuity for people with severe mental illness leads to better health outcomes, reduced hospital admission, and is more cost‐effective [30]. A previous study has called for higher level monitoring of EIP SUs for at least 1‐year post‐discharge to extend continuity of care [31]. By following SUs through and after discharge, our study supports this previous research, highlighting their desire for regular, proactive care to support their journey, both in terms of relationship‐based care (at the psycho‐social level) and greater coordination of care (at the practical level). If SUs receive proactive, personalised care after discharge ‐ elements of care they associate with EIP ‐ our research shows that they report more positive experiences.

Our study also adds to research calling for greater collaboration across the primary and specialist care interface, both in general terms [32, 33] and specifically in relation to transitions from EIP [34, 35].

### 6.3 Strengths and Limitations

This study includes diverse SUs and carers in terms of demographics, experiences and outcomes. The longitudinal component allowed us to follow SU journeys through the discharge process and into the post‐discharge period. We used a range of recruitment methods to ensure the sample was as inclusive as possible.

A key strength of this study is the meaningful involvement of SUs and carers in its design and analysis. Two lived experience co‐investigators (authors V.G. and D.S.) and the EXTEND‐ing service user and carer advisory group contributed throughout, formulating topic guides and recruitment materials, and meeting with the research team regularly to discuss data and identify emerging themes. One limitation of the PPIE involvement was the lack of participation of EXTEND‐ing members in the academic writing process. Our lived experience co‐investigators both contributed to this paper, but EXTEND‐ing members did not respond to the invitation to comment on the paper. In the future, we will explore other ways of involving people with lived experience in writing academic papers.

There are other limitations to this study. Only three of the sixteen SUs were discharged to CMHT, resulting in greater focus on transitions from EIP to primary care. SUs who remained unwell at discharge and were transferred to CMHTs are less likely to have participated in our study. Our sample demographics may not exactly represent the EIP population (women were over‐represented), so generalisability may be limited as men tend to have poorer outcomes from psychotic illness [36, 37]. There are opportunities for future research exploring the perspectives of male SUs and SUs discharged to CMHTs.

### 6.4 Implications for Practice

This study highlights the importance of involving SUs and carers in decision‐making and planning around EIP discharge. We suggest that collaboration is needed between SUs, carers and primary care while SUs are under EIP, to maintain relationships and support effective discharge. Proactive contact from primary care to the service user is needed around the point of discharge and in the early post‐discharge period. A joint primary care consultation at point of discharge, involving the service user, carer if appropriate, EIP care coordinator and GP, could aid the transition. Mental health practitioners embedded within practices could play a key role in supporting this transition [38].

Primary care could also assist with signposting to relevant voluntary sector and community organisations and networks. Our findings show that carer needs in particular are often overlooked; clinicians could utilise the ‘carers register’ and proactively offer guidance about sources of support for carers of people under EIP and discharged from EIP.

## 7 Conclusion

This longitudinal qualitative study fills the research gap around the perspectives of service users and carers discharged from EIP services, exploring their journey through discharge and into the early period of post‐discharge care. It highlights the importance of improved collaboration between healthcare practitioners and SUs and carers, and the value of relationship‐based care. We have also provided practical suggestions about the role that primary care could play in supporting SUs and carers during and after discharge from EIP.

## Author Contributions

**Michelle Rickett:** methodology, investigation, writing – original draft, formal analysis, writing – review and editing, data curation, validation. **Tom Kingstone:** conceptualization, methodology, validation, writing – original draft, funding acquisition, investigation, formal analysis, supervision, writing – review and editing. **Veenu Gupta:** investigation, funding acquisition, writing – original draft, validation, writing – review and editing, methodology. **David Shiers:** conceptualization, investigation, funding acquisition, writing – original draft, writing – review and editing, validation, methodology. **Paul French:** investigation, funding acquisition, writing – review and editing. **Belinda Lennox:** investigation, funding acquisition, writing – review and editing. **Ed Penington:** writing – review and editing, validation. **Ryan Williams:** writing – review and editing, validation. **Isobel Hoppe:** writing – review and editing, formal analysis. **Carolyn A. Chew‐Graham:** conceptualization, investigation, funding acquisition, methodology, validation, writing – review and editing, formal analysis, project administration, data curation, supervision.

## Ethics Statement

Research ethics approval was obtained in September 2022 from the North of Scotland Research Ethics Committee (reference [22]/NS/0113) and Health Research Authority (Integrated Research Application System ID: 313927).

## Conflicts of Interest

The authors declare no conflicts of interest.

## Supporting information

supmat.

Supplementary materials ‐ Topic guides.

## Data Availability

Anonymised data is available on reasonable request. The data that support the findings of this study are available on request from the corresponding author. The data are not publicly available due to privacy or ethical restrictions.

## References

[1] M. Marshall , S. Lewis , A. Lockwood , R. Drake , P. Jones , and T. Croudace , “Association Between Duration of Untreated Psychosis and Outcome in Cohorts of First‐Episode Patients: A Systematic Review,” Archives of General Psychiatry 62, no. 9 (2005): 975–983. 16143729 10.1001/archpsyc.62.9.975

[2] C. U. Correll , B. Galling , A. Pawar , et al., “Comparison of Early Intervention Services vs Treatment as Usual for Early‐Phase Psychosis: A Systematic Review, Meta‐Analysis, and Meta‐Regression,” JAMA Psychiatry 75, no. 6 (2018): 555–565. 29800949 10.1001/jamapsychiatry.2018.0623 PMC6137532

[3] S. Puntis , A. Minichino , F. De Crescenzo , et al., “Specialised Early Intervention Teams for Recent‐Onset Psychosis,” Cochrane Database of Systematic Reviews 11, no. 11 (2020): CD013288, 10.1002/14651858.CD013288.pub2. 33135811 PMC8092671

[4] K. Barr , J. Ormrod , and R. Dudley , “An Exploration of What Service Users Value About Early Intervention in Psychosis Services,” Psychology and Psychotherapy: Theory, Research and Practice 88 (2015): 468–480, 10.1111/papt.12051. 25572755

[5] R. Williams , A. Morris , V. Gupta , et al., “Predictors of Positive Patient‐Reported Outcomes From ‘Early Intervention in Psychosis’: A National Cross‐Sectional Study,” BMJ Mental Health 26 (2023): e300716. 10.1136/bmjment-2023-300716 PMC10577709 37541700

[6] J. Allard , S. Lancaster , S. Clayton , T. Amos , and M. Birchwood , “Carers’ and Service Users’ Experiences of Early Intervention in Psychosis Services: Implications for Care Partnerships,” Early Intervention in Psychiatry 12, no. 3 (2018): 410–416, 10.1111/eip.12309. 26758476

[7] M. Loughlin , K. Berry , J. Brooks , and S. Bucci , “Moving on From Early Intervention for Psychosis Services: Service User Perspectives on the Facilitators and Barriers of Transition,” Early Intervention in Psychiatry 13, no. 6 (2019): 1396–1403. 30672143 10.1111/eip.12780

[8] S. Puntis , S. Pappa , and B. Lennox , “What Happens After Early Intervention? Mapping Early Intervention in Psychosis Care Pathways in the 12 Months After Discharge,” Early Intervention in Psychiatry 18, no. 1 (2024): 49–57. 37220964 10.1111/eip.13433

[9] H. Lester , N. Khan , P. Jones , et al., “Service Users’ Views of Moving on From Early Intervention Services for Psychosis: A Longitudinal Qualitative Study in Primary Care,” British Journal of General Practice 62, no. 596 (2012): e183–e190, 10.3399/bjgp12X630070. PMC3289824 22429435

[10] S. Woodward , S. Bucci , D. Edge , and K. Berry , “Barriers and Facilitators to ‘Moving on’ From Early Intervention in Psychosis Services,” Early Intervention in Psychiatry 13, no. 4 (2019): 914–921, 10.1111/eip.12708. 30051626

[11] M. Rickett , T. Kingstone , V. Gupta , et al., “Collaboration Across the Primary and Specialist Care Interface in Early Intervention in Psychosis Services: A Qualitative Study,” British Journal of General Practice: The Journal of the Royal College of General Practitioners 74, no. 747 (2024): 709, 10.3399/BJGP.2023.0558. PMC11350604 38499296

[12] D. Sud , E. Bradley , J. Tritter , and I. Maidment , “The Impact of Providing Care for Physical Health in Severe Mental Illness on Informal Carers: A Qualitative Study,” BMC Psychiatry 24 (2024): 426, 10.1186/s12888-024-05864-3. 38844879 PMC11154995

[13] J. Rowe , “Great Expectations: A Systematic Review of the Literature on the Role of Family Carers in Severe Mental Illness, and Their Relationships and Engagement With Professionals,” Journal of Psychiatric and Mental Health Nursing 19 (2012): 70–82, 10.1111/j.1365-2850.2011.01756.x. 22070436

[14] J. Mulligan , W. Sellwood , G. S. Reid , S. Riddell , and N. Andy , “Informal Caregivers in Early Psychosis: Evaluation of Need for Psychosocial Intervention and Unresolved Grief,” Early Intervention in Psychiatry 7 (2013): 291–299, 10.1111/j.1751-7893.2012.00369.x. 22741743

[15] J. Onwumere , D. Shiers , and C. Chew‐Graham , “Understanding the Needs of Carers of People With Psychosis in Primary Care,” British Journal of General Practice 66, no. 649 (2016): 400–401, 10.3399/bjgp16X686209. PMC4979915 27481960

[16] A. Lavis , H. Lester , L. Everard , et al., “Layers of Listening: Qualitative Analysis of the Impact of Early Intervention Services for First‐Episode Psychosis on Carers’ Experiences,” British Journal of Psychiatry 207, no. 2 (2015): 135–142, 10.1192/bjp.bp.114.146415. 25999336

[17] M. Rickett , T. Kingstone , V. Gupta , et al., “Collaboration Across the Primary and Specialist Care Interface in Early Intervention in Psychosis Services: A Qualitative Study,” British Journal of General Practice: The Journal of the Royal College of General Practitioners 74, no. 747 (2024): 709, 10.3399/BJGP.2023.0558. PMC11350604 38499296

[18] M. Crotty , The Foundations of Social Research: Meaning and Perspective in the Research Process (Sage Publications, 2015).

[19] D. Ridge , L. Bullock , H. Causer , et al., “Imposter Participants’ in Online Qualitative Research: A New and Increasing Threat to Data Integrity?,” Health Expectations 26, no. 3 (2023): 941–944. 36797816 10.1111/hex.13724 PMC10154888

[20] V. Braun and V. Clarke , “Using Thematic Analysis in Psychology,” Qualitative Research in Psychology 3 (2006): 77–101, 10.1191/1478088706qp063oa.

[21] J. Creswell , “Data Analysis and Representation,” in Qualitative Inquiry and Research Design: Choosing Among Five Approaches, 2nd ed., eds. J. Creswell (Sage, 2007), 179–212.

[22] K. Malterud , V. D. Siersma , and A. D. Guassora , “Sample Size in Qualitative Interview Studies Guided by Information Power,” Qualitative Health Research 26, no. 13 (2016): 1753–1760. 26613970 10.1177/1049732315617444

[23] V. Braun , V. Clarke , N. Hayfield , et al., “Doing Reflexive Thematic Analysis,” in Supporting Research in Counselling and Psychotherapy: Qualitative, Quantitative, and Mixed Methods Research, eds. S. Bager‐Charleson , A. McBeath (Springer International, 2023), 19–38.

[24] M. Loughlin , K. Berry , J. Brooks , and S. Bucci , “Moving on From Early Intervention for Psychosis Services: Service User Perspectives on the Facilitators and Barriers of Transition,” Early Intervention in Psychiatry 13, no. 6 (2019): 1396–1403. 30672143 10.1111/eip.12780

[25] H. Lester , N. Khan , P. Jones , et al., “Service Users’ Views of Moving on From Early Intervention Services for Psychosis: A Longitudinal Qualitative Study in Primary Care,” British Journal of General Practice 62, no. 596 (2012): e183–e190, 10.3399/bjgp12X630070. PMC3289824 22429435

[26] J. Allard , S. Lancaster , S. Clayton , T. Amos , and M. Birchwood , “Carers’ and Service Users’ Experiences of Early Intervention in Psychosis Services: Implications for Care Partnerships,” Early Intervention in Psychiatry 12, no. 3 (2018): 410–416, 10.1111/eip.12309. 26758476

[27] J. Onwumere , D. Shiers , and C. Chew‐Graham , “Understanding the Needs of Carers of People With Psychosis in Primary Care,” British Journal of General Practice 66, no. 649 (2016): 400–401, 10.3399/bjgp16X686209. PMC4979915 27481960

[28] R. Williams , E. G. Ostinelli , J. Agorinya , et al., “Comparing Interventions for Early Psychosis: A Systematic Review and Component Network Meta‐Analysis,” EClinicalMedicine 70, no. 70 (2024): 102537, 10.1016/j.eclinm.2024.102537. 38516103 PMC10955207

[29] H. Lester , M. Birchwood , S. Bryan , E. England , H. Rogers , and N. Sirvastava , “Development and Implementation of Early Intervention Services for Young People With Psychosis: Case Study,” British Journal of Psychiatry 194, no. 5 (2009): 446–450, 10.1192/bjp.bp.108.053587. 19407276

[30] C. E. Adair , G. M. McDougall , C. R. Mitton , et al., “Continuity of Care and Health Outcomes Among Persons With Severe Mental Illness,” Psychiatric Services 56, no. 9 (2005): 1061–1069, 10.1176/appi.ps.56.9.1061. 16148318

[31] S. M. Kam , S. P. Singh , and R. Upthegrove , “What Needs to Follow Early Intervention? Predictors of Relapse and Functional Recovery Following First‐Episode Psychosis,” Early Intervention in Psychiatry 9, no. 4 (2015): 279–283, 10.1111/eip.12099. 24251970

[32] P. Aggarwal and H. P. Patel , “Gatekeepers, Wizards, and a Mutual Appreciation of Each Other's Kingdoms,” BMJ (Clinical Research ed.) 375 (2021): n3005, 10.1136/bmj.n3005. 34906984

[33] Academy of Medical Royal Colleges . General Practice and Secondary Care: Working Better Together, (2023), accessed November 27, 2024, https://www.aomrc.org.uk/wp-content/uploads/2023/05/GPSC_Working_better_together_0323.pdf.

[34] M. Rickett , T. Kingstone , V. Gupta , et al., “Collaboration Across the Primary and Specialist Care Interface in Early Intervention in Psychosis Services: A Qualitative Study,” British Journal of General Practice: The Journal of the Royal College of General Practitioners 74, no. 747 (2024): 709, 10.3399/BJGP.2023.0558. PMC11350604 38499296

[35] S. Woodward , S. Bucci , D. Edge , and K. Berry , “Barriers and Facilitators to ‘Moving On’ From Early Intervention in Psychosis Services,” Early Intervention in Psychiatry 13 (2019): 914–921, 10.1111/eip.12708. 30051626

[36] Royal College of Psychiatrists . National Clinical Audit of Psychosis: Audit Reports, (2022), https://www.rcpsych.ac.uk/improving-care/ccqi/national-clinical-audits/national-clinical-audit-of-psychosis/audit-reports).

[37] L. S. Grossman , M. Harrow , C. Rosen , R. Faull , and G. P. Strauss , “Sex Differences in Schizophrenia and Other Psychotic Disorders: A 20‐Year Longitudinal Study of Psychosis and Recovery,” Comprehensive Psychiatry 49, no. 6 (2008): 523–529, 10.1016/j.comppsych.2008.03.004. 18970899 PMC2592560

[38] NHS England . Expanding Our Workforce, https://www.england.nhs.uk/gp/expanding-our-workforce/, accessed 27.11.24.

